# Efficacy and Safety of Pelubiprofen for Primary Dysmenorrhea: A Multicenter, Randomized, Double-Blind, Placebo-Controlled, Two-Period Crossover Trial

**DOI:** 10.3390/jcm15072658

**Published:** 2026-03-31

**Authors:** Joo Hyun Park, Inha Lee, Sung Pil Choo, Jae-Hoon Lee, Seung Hwa Hong, Jong Kil Joo, Hyun Tae Park, Mi Ran Kim, Dong-Yun Lee, Kyong Wook Yi, Sang Ho Yoon, Jung-Ho Shin, Jung Ryeol Lee, Sung Hoon Kim, SiHyun Cho, Young Sik Choi

**Affiliations:** 1 Department of Obstetrics and Gynecology, Yongin Severance Hospital, Yonsei University College of Medicine, Yongin 16995, Republic of Korea; beanpearl@yuhs.ac; 2 Institute of Women’s Life Medical Science, Yonsei University College of Medicine, Seoul 03722, Republic of Korea; 3 Department of Obstetrics and Gynecology, Gangnam Severance Hospital, Yonsei University College of Medicine, Seoul 06273, Republic of Korea; 4 Department of Obstetrics and Gynecology, Inha University Hospital, College of Medicine, Inha University, Incheon 22332, Republic of Korea; 5 Department of Obstetrics and Gynecology, Chungbuk National University Hospital, Cheongju 28644, Republic of Korea; 6 Department of Obstetrics and Gynecology, Biomedical Research Institute, Pusan National University Hospital, Pusan National University School of Medicine, Busan 49241, Republic of Korea; 7 Department of Obstetrics and Gynecology, Korea University College of Medicine, Seoul 02841, Republic of Korea; 8 Department of Obstetrics and Gynecology, Ajou University School of Medicine, Suwon 16499, Republic of Korea; 9 Department of Obstetrics and Gynecology, Samsung Medical Center, Sungkyunkwan University School of Medicine, Seoul 06351, Republic of Korea; 10 Department of Obstetrics and Gynecology, Dongguk University Medical College, Goyang 10326, Republic of Korea; yoonprou@dumc.or.kr; 11 Department of Obstetrics and Gynecology, Seoul National University College of Medicine, Seoul 03080, Republic of Korea; 12 Department of Obstetrics & Gynecology, Asan Medical Center, University of Ulsan College of Medicine, Seoul 05505, Republic of Korea; 13 Department of Obstetrics and Gynecology, Severance Hospital, Yonsei University College of Medicine, Seoul 03722, Republic of Korea

**Keywords:** primary dysmenorrhea, pelubiprofen, nonsteroidal anti-inflammatory drugs, randomized controlled trial, crossover study, TOTPAR, SPID, menstrual pain, analgesic efficacy

## Abstract

**Background:** Primary dysmenorrhea is a common gynecologic condition that frequently requires pharmacologic treatment. Pelubiprofen, a 2-arylpropionic acid-derived prodrug with relatively selective cyclooxygenase-2 inhibitory activity, has demonstrated analgesic efficacy in acute pain conditions. **Methods:** This multicenter, randomized, double-blind, placebo-controlled, crossover phase 3 trial randomized 120 women aged 19–44 years with primary dysmenorrhea to one of two treatment sequences over two menstrual cycles. Pelubiprofen at 45 mg or a matching placebo was administered at the onset of moderate or severe menstrual pain. The co-primary endpoints were time-weighted sums of the total pain relief (TOTPAR-8) and pain intensity difference (SPID-8) during the first 8 h after dosing. **Results:** Of 120 randomized women, 115 comprised the modified intention-to-treat population and 116 comprised the safety population. Pelubiprofen demonstrated significantly greater analgesic efficacy than placebo, with least-squares mean TOTPAR-8 values of 22.17 versus 15.50 and SPID-8 values of 10.00 versus 6.17 (both *p* < 0.0001). Significant between-treatment differences were also observed at 12 h (TOTPAR-12 and SPID-12). Treatment-emergent adverse events occurred in 9/113 (8.0%) pelubiprofen treatment periods and 10/112 (8.9%) placebo treatment periods; all events were mild to moderate, and the only serious adverse event occurred during a placebo treatment period and was judged to be unrelated to study treatment. **Conclusions:** Pelubiprofen at 45 mg provided superior short-term analgesic efficacy compared with placebo and was generally well tolerated in women with primary dysmenorrhea.

## 1. Introduction

Primary dysmenorrhea is characterized by cramping lower abdominal pain during menstruation in the absence of identifiable pelvic pathology [1,2]. It is frequently accompanied by nausea, fatigue, headache, and diarrhea and can substantially impair daily functioning, work productivity, and quality of life [3]. Reported prevalence estimates range from approximately 50% to 90% among menstruating women, and a considerable proportion experience symptoms that are moderate to severe and require pharmacologic treatment [1,4].

Pathophysiologically, excessive uterine prostaglandin production during menstruation—particularly prostaglandin F2α—has been implicated. Elevated prostaglandins increase myometrial contractions and intrauterine pressure and reduce uterine blood flow, leading to uterine ischemia and nociceptor sensitization; these mechanisms are considered central to menstrual pain and related systemic symptoms [1,2,3].

Nonsteroidal anti-inflammatory drugs (NSAIDs) inhibit cyclooxygenase-mediated prostaglandin synthesis and are therefore recommended as first-line pharmacologic therapy for primary dysmenorrhea [5]. Randomized trials and systematic reviews support the effectiveness of commonly used NSAIDs such as ibuprofen and naproxen [5,6]. Hormonal contraceptives can also alleviate symptoms, particularly when longer-term cycle control is desired, but NSAIDs remain the most commonly used initial option [2,5]. Despite established benefit, repeated use of conventional non-selective NSAIDs may be limited by gastrointestinal adverse events (e.g., dyspepsia, mucosal injury, ulceration, and bleeding) and potential cardiovascular risks, motivating the development of agents with improved tolerability and/or greater COX-2 selectivity [7,8].

Pelubiprofen is a 2-arylpropionic acid-derived NSAID structurally related to ibuprofen with prodrug characteristics and relatively selective COX-2 inhibitory activity, which has been developed to improve gastrointestinal safety [9]. Its analgesic efficacy and tolerability have been reported in inflammatory and pain conditions such as rheumatoid arthritis and osteoarthritis [10,11], whereas evidence from well-controlled clinical trials in primary dysmenorrhea remains limited. We therefore conducted a phase 3, multicenter, randomized, double-blind, placebo-controlled, two-period crossover trial to evaluate the efficacy and safety of pelubiprofen in women with moderate-to-severe primary dysmenorrhea. The primary objective was to compare pelubiprofen with placebo for cumulative pain relief and pain intensity reduction over the first 8 h after dosing; the secondary objectives included evaluating outcomes through 12 h and patient-reported global satisfaction, together with overall tolerability.

## 2. Materials and Methods

### 2.1. Study Design

This phase 3 study was a multicenter, randomized, double-blind, placebo-controlled, two-period crossover trial conducted in women with clinically diagnosed primary dysmenorrhea who were screened to exclude suspected secondary causes. The trial was performed in outpatient clinics at 12 centers in the Republic of Korea. Study activities took place from October 2020 to July 2022, and enrollment occurred between October 2021 and May 2022.

**Figure 1:**
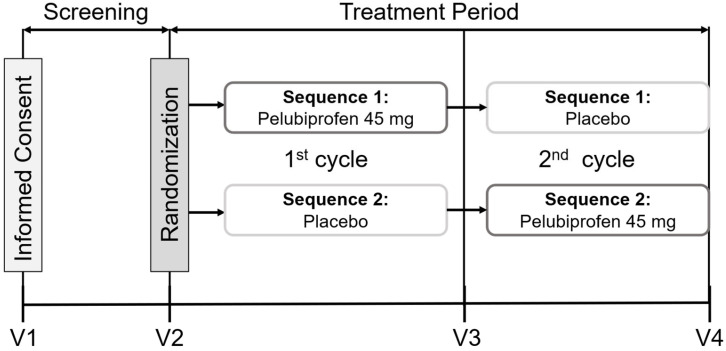
Study design and treatment sequences in the two-period crossover trial.

The protocol was conducted in compliance with Korean Good Clinical Practice (KGCP), ICH Good Clinical Practice (ICH-GCP), and the Declaration of Helsinki. Institutional Review Board approval was obtained from Severance Hospital (IRB No. 4-2020-0968), and all participants provided written informed consent before any study-related procedure. The trial was registered at ClinicalTrials.gov (NCT04541134). The study design and treatment sequences are presented in Figure 1.

### 2.2. Participants

Eligibility was assessed at screening. Women aged 19–44 years who were in good general health, had regular menstrual cycles (28 ± 7 days) over the preceding year, and reported moderate-to-severe primary dysmenorrhea requiring analgesics in at least four of the previous six menstrual cycles were eligible.

Moderate dysmenorrhea was defined as pain that interfered with daily activities but was generally relieved by analgesics, whereas severe dysmenorrhea was defined as pain that disrupted daily activities.

Key exclusion criteria included pregnancy or breastfeeding; oral contraceptive use within 12 weeks before study entry (unless moderate-to-severe dysmenorrhea persisted despite stable contraceptive use); suspected or confirmed secondary dysmenorrhea or reproductive tract disease; and use of concomitant medications that could confound pain assessment. Secondary dysmenorrhea was excluded based on protocol-defined clinical screening criteria and investigator assessment. Gynecologic ultrasonography was performed using standard equipment available at each study site only when secondary dysmenorrhea was clinically suspected at screening or when a result obtained within 8 weeks was available; however, standardized pelvic imaging, including transvaginal ultrasonography, was not mandated for all participants across study sites. Systemic analgesics and NSAIDs were prohibited from 3 days before study drug administration through Pain Day 3, including long-acting agents such as meloxicam and piroxicam; sedatives, muscle relaxants, antispasmodics, tricyclic antidepressants, and neuroleptics were prohibited from 2 days before dosing through Pain Day 3.

### 2.3. Randomization and Blinding

The trial used a two-sequence, two-period crossover design over two menstrual cycles. Participants were allocated 1:1 to one of the two treatment sequences according to a computer-generated randomization schedule. An independent statistician generated the allocation list using SAS^®^ 9.4 (SAS Institute Inc., Cary, NC, USA) and remained blinded to participant identifiers and clinical information.

To maintain double blinding, pelubiprofen and placebo tablets were visually indistinguishable. Emergency unblinding was permitted only if knowledge of the assigned treatment was considered essential for clinical management. No unblinding occurred during the study.

### 2.4. Treatment Protocol

Participants completed two treatment cycles according to their assigned sequence, receiving pelubiprofen at 45 mg and a matching placebo in alternate cycles. The 45 mg pelubiprofen tablets and matching placebo were supplied by Daewon Pharmaceutical Co., Ltd. (Seoul, Republic of Korea) and were identical in appearance.

Pain Day 1 was defined as the first onset of moderate or severe menstrual pain after the start of menstruation. At that time, participants took one oral dose of the assigned study medication. If additional analgesia was needed, one additional dose of the assigned study medication was allowed only after completion of the 8 h assessment on Pain Day 1. On Days 2 and 3, participants could take the assigned study medication as needed up to twice daily (maximum daily dose equivalent to 90 mg pelubiprofen); dosing was not required in the absence of pain.

To ensure completion of scheduled post-dose efficacy assessments, cycles in which qualifying pain began after 18:00 on Pain Day 1 were not treated. For each cycle, study medication could be taken for up to 3 consecutive days.

After completing Pain Day 1, participants contacted the study coordinator to arrange a follow-up visit within 14 days after Pain Day 3 of that treatment cycle, at which medication for the subsequent cycle was dispensed. When treatment initiation was not feasible (e.g., no qualifying pain, intercurrent illness, travel, or pain onset after 18:00), the cycle could be postponed for up to four menstrual cycles. Participants who did not take study medication in two consecutive cycles were considered dropouts.

Participants recorded menstrual pain intensity immediately before any additional dosing on Days 1–3. On Pain Day 1, pain relief and pain intensity were assessed using a diary at 0.25, 0.5, 0.75, 1, 1.5, 2, 3, 4, 5, 6, 7, 8, 9, 10, 11, and 12 h after the first dose. Pain relief was rated on a 0–4 scale (0 = no relief; 4 = complete relief), and pain intensity was rated on a 0–3 scale (0 = none; 3 = severe). On Days 2 and 3, the maximum pain intensity was recorded at bedtime using the same scale. All evaluations were recorded in the diary.

### 2.5. Outcome Measures

Because pain is subjective and varies over time, both cumulative and time-specific endpoints were used. The co-primary endpoints were cumulative total pain relief over the first 8 h (TOTPAR-8) and cumulative pain intensity reduction over the same interval (SPID-8).

Pain relief (0–4) and pain intensity (0–3) were collected at predefined post-dose time points. The pain intensity difference (PID) at each time point was calculated as the baseline pain intensity score minus the corresponding post-dose pain intensity score.

The secondary endpoints included TOTPAR and SPID through 12 h (TOTPAR-12 and SPID-12), time-specific pain relief scores, and time-specific PID values. The composite metric PRID (pain relief plus the corresponding PID at each assessment) was calculated and summarized over 8 and 12 h (SPRID-8 and SPRID-12). Global satisfaction with the study medication was rated on a 5-point scale (0 = poor; 1 = fair; 2 = good; 3 = very good; 4 = excellent) at 12 h after the first dose on Day 1 and again at bedtime on Days 2 and 3 of each cycle.

### 2.6. Safety Assessments

Safety evaluations included physical examinations, vital signs, standard laboratory tests, and a 12-lead electrocardiogram. Adverse events were defined as any untoward medical occurrence—including symptoms, signs, or clinically meaningful abnormalities in laboratory or physical findings—that newly appeared or worsened during the study, irrespective of assessed causality. Investigators documented each event and evaluated its severity and relationship with the study medication.

### 2.7. Statistical Analysis

The sample size required to detect differences between pelubiprofen and placebo in TOTPAR-8 and SPID-8 was determined. Based on assumed between-treatment mean differences of 5.23 (SD 10.23) for TOTPAR-8 and 3.64 (SD 7.19) for SPID-8, 84 participants were required to provide 90% power with a two-sided α of 0.05. Allowing for an anticipated 30% dropout rate, 120 participants were planned.

Baseline characteristics are summarized for all randomized participants. Continuous variables (age, height, weight, body mass index, and the number of dysmenorrhea episodes during the six menstrual cycles before screening) were compared between sequences using a two-sample t test. Dysmenorrhea severity categories were compared using Pearson’s chi-square test.

Efficacy analyses were primarily conducted in the modified intention-to-treat (mITT) population, with sensitivity analyses in the per-protocol (PP) population. The mITT population included randomized participants who received at least one dose of study medication and had at least one post-dose assessment of pain relief or pain intensity within 8 h after the first dose on Day 1. Participants were excluded from the mITT analysis if neither pain relief nor pain intensity was assessed at the first scheduled post-dose time point (0.25 h) or if two or more consecutive values were missing within the first 2 h.

TOTPAR, SPID, and summed PRID endpoints were calculated as time-weighted sums. Between-treatment effects were estimated using an analysis of covariance model with treatment, treatment sequence, and cycle as fixed effects; participant as a random effect; and period-specific baseline pain intensity as a covariate. Baseline pain intensity was defined as the pre-dose pain intensity measured on Day 1 of each treatment cycle. Two co-primary endpoints were prespecified, and study success was defined a priori only if both endpoints achieved statistical significance; therefore, no additional multiplicity adjustment was applied. Other continuous efficacy outcomes were analyzed using the same model. Global satisfaction was analyzed using generalized estimating equations with treatment, treatment sequence, and cycle as factors and period-specific baseline pain intensity as a covariate.

Missing efficacy data in the mITT and PP populations were handled using last observation carried forward, except when data were missing at the first post-dose assessment or when two or more consecutive values were missing within the first 2 h (in which case values were not imputed). Safety analyses were conducted in the safety population (participants who received at least one dose of study medication). Adverse events were coded using the Medical Dictionary for Regulatory Activities (version 25.0) and summarized descriptively. Analyses were performed using SAS^®^ 9.4, and statistical significance was defined as a two-sided *p* value < 0.05. Data management and statistical analyses were performed by a sponsor-contracted CRO according to the predefined analysis plan, and the accuracy of the outputs was verified through quality-control procedures.

## 3. Results

### 3.1. Patient Disposition

**Figure 2:**
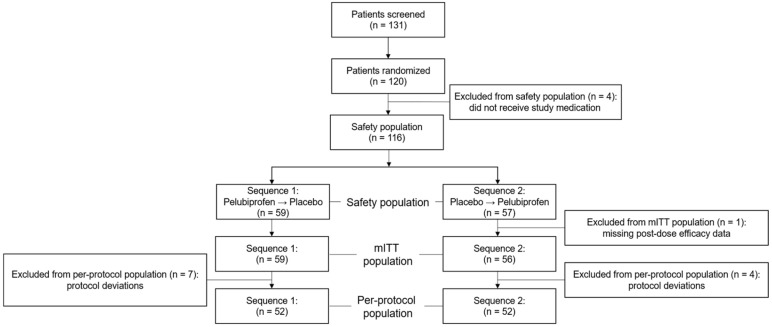
CONSORT flow diagram of patient disposition.

Participant flow and analysis populations are summarized in Figure 2. Screening was completed in 131 women, and 120 were randomized to Sequence 1 or Sequence 2. A total of 4 randomized women did not receive any study medication; therefore, 116 women comprised the safety population (Sequence 1, *n* = 59; Sequence 2, *n* = 57).

The modified intention-to-treat (mITT) population included 115 women who received at least one dose of study medication and had at least one post-dose assessment of pain relief or pain intensity within 8 h after the first dose on Day 1. One woman in Sequence 2 was excluded because both pain relief and pain intensity had two or more consecutive missing values within the first 2 h after dosing on Day 1, resulting in 59 and 56 women in Sequences 1 and 2, respectively. A sensitivity analysis including this participant using multiple imputation yielded results consistent with the primary analysis. After excluding major protocol deviations (e.g., dispensing errors or insufficient efficacy data), 104 women (52 per sequence) constituted the per-protocol (PP) population.

Because this was a two-period crossover trial, counts by treatment reflect evaluable treatment periods. In the safety population, 112 placebo periods and 113 pelubiprofen periods were included in the analysis. The corresponding numbers in the mITT population were 111 (placebo) and 113 (pelubiprofen), whereas the PP population included 104 periods in each treatment condition. The mITT population was used for primary efficacy analyses, and PP analyses were conducted as sensitivity analyses.

### 3.2. Baseline Characteristics

Baseline demographic and dysmenorrhea-related characteristics were balanced between the two sequences (Table 1). The mean age was 30.97 ± 5.48 years in Sequence 1 and 29.17 ± 4.89 years in Sequence 2 (*p* = 0.060). Anthropometric measures were comparable, including height (161.82 ± 4.68 vs. 161.86 ± 5.29 cm), body weight (56.10 ± 7.01 vs. 56.42 ± 6.16 kg), and body mass index (21.48 ± 3.01 vs. 21.55 ± 2.32 kg/m^2^) (all *p* ≥ 0.792).

The mean number of dysmenorrhea episodes during the six menstrual cycles before screening was 5.65 ± 0.68 in Sequence 1 and 5.62 ± 0.74 in Sequence 2 (*p* = 0.798). Moderate dysmenorrhea was reported by 83.33% (50/60) and 86.67% (52/60) of participants in Sequences 1 and 2, respectively, and the remaining participants reported severe dysmenorrhea (*p* = 0.609). Overall, no baseline variable differed significantly between sequences.

### 3.3. Primary Efficacy Outcomes

Primary efficacy was assessed in the modified intention-to-treat (mITT) population. Pelubiprofen was associated with higher values than placebo for both co-primary endpoints during the first 8 h after the initial dose (Table 2). The estimated treatment contrast (pelubiprofen minus placebo) was 6.67 for TOTPAR-8 (95% CI 4.82–8.53; *p* < 0.0001) and 3.82 for SPID-8 (95% CI 2.71–4.94; *p* < 0.0001). Model-estimated least-squares (LS) means ± SE were 22.17 ± 0.75 versus 15.50 ± 0.76 for TOTPAR-8 and 10.00 ± 0.44 versus 6.17 ± 0.45 for SPID-8 (pelubiprofen vs. placebo).

The between-treatment separation was also evident at 12 h (Table 2). Pelubiprofen exceeded placebo by 8.54 for TOTPAR-12 (95% CI 5.81–11.27; *p* < 0.0001) and by 4.72 for SPID-12 (95% CI 3.13–6.31; *p* < 0.0001). The corresponding LS means ± SE were 33.95 ± 1.14 versus 25.41 ± 1.15 for TOTPAR-12 and 15.34 ± 0.67 versus 10.62 ± 0.67 for SPID-12. Because the summed endpoints were derived from ordinal scales and the assumption of normality was not consistently met (assessed via the Shapiro–Wilk test), an additional rank-based ANCOVA sensitivity analysis was conducted. The rank-based analysis yielded results consistent with the primary analysis, confirming the stability of the findings. Furthermore, additional doses were permitted only after 8 h from the initial dose. On Day 1, additional dosing between 8 and 12 h occurred in 18.92% (21/111) of the placebo periods and 14.16% (16/113) of the pelubiprofen periods. A sensitivity analysis adjusting for the total dose taken per cycle for endpoints beyond 8 h showed results consistent with the primary analysis, confirming the robustness of the efficacy findings. In an additional ad hoc analysis, no significant carryover effect was observed for either TOTPAR-8 (*p* = 0.4565) or SPID-8 (*p* = 0.9147), suggesting that treatment administered in the previous cycle did not materially influence the subsequent cycle.

### 3.4. Secondary Efficacy Outcomes

**Figure 3:**
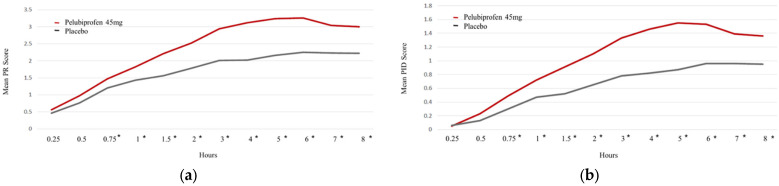
Time-course of pain relief and pain intensity difference (PID) during the first 8 h after the initial dose (mITT population). Asterisks indicate *p* < 0.05 for pelubiprofen versus placebo at the corresponding time point: (**a**) pain relief; (**b**) PID.

Time-course analyses on Pain Day 1 showed early and sustained separation between treatments. From 0.75 h after dosing onward, both the pain intensity difference (PID) and pain relief scores were consistently higher during the pelubiprofen period than during the placebo period, and this pattern continued through 8 h (Figure 3).

**Figure 4:**
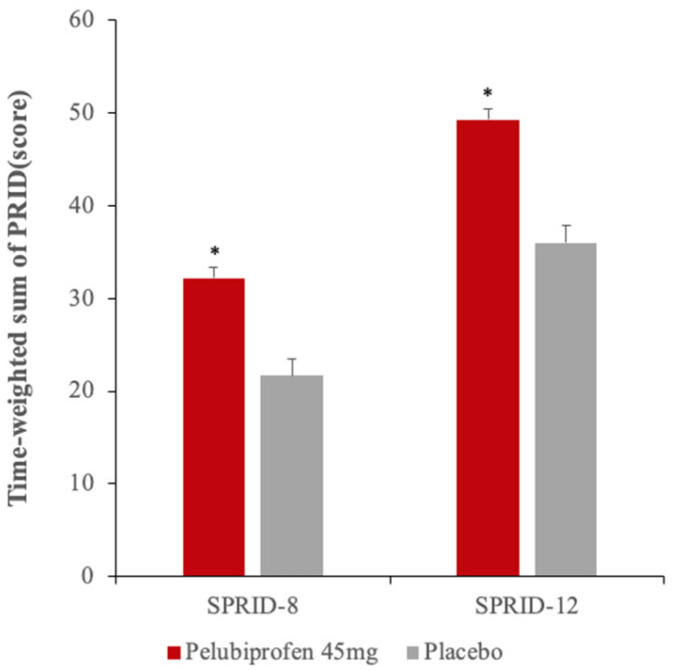
SPRID-8 and SPRID-12 in the mITT population. Bars represent LS mean (±SE) estimates from the ANCOVA model. * *p* < 0.0001 versus placebo. Abbreviations: SPRID, summed pain relief and intensity difference; LS, least squares; SE, standard error; ANCOVA, analysis of covariance.

When pain relief and PID were combined into PRID-based composite summaries and integrated over time, pelubiprofen again demonstrated greater efficacy than placebo. Both SPRID-8 and SPRID-12 were significantly higher during the pelubiprofen period than during the placebo period (Figure 4).

**Figure 5:**
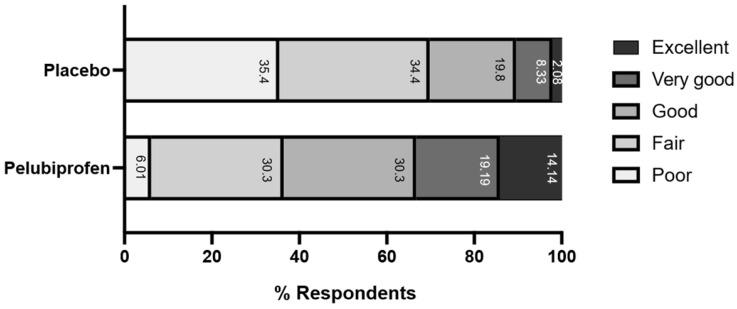
Distribution of participants’ global evaluation of the study medication at 12 h on Day 1 (mITT population). Bars show the proportion of responses in each category (poor, fair, good, very good, and excellent).

Global satisfaction ratings also favored pelubiprofen (Figure 5). At 12 h on Day 1, the mean ± SD global satisfaction score was 2.08 ± 1.22 with pelubiprofen and 1.07 ± 1.08 with placebo. On Day 1, 63.64% of participants selected “good,” “very good,” or “excellent” during the pelubiprofen period, compared with 30.21% during the placebo period. The proportion reporting ratings of “good” or higher remained numerically higher with pelubiprofen on Day 2 (55.26% [21/38] vs. 25.93% [7/27]) and Day 3 (83.33% [10/12] vs. 44.44% [4/9]). The between-treatment difference reached statistical significance on Day 2 (mean difference 1.23 ± 0.57; *p* = 0.0305).

Between-treatment contrasts in maximum pain intensity recorded at bedtime on Days 2 and 3 were small and not statistically significant. The estimated pelubiprofen–placebo differences were −0.02 ± 0.09 on Day 2 (*p* = 0.8651) and −0.03 ± 0.07 on Day 3 (*p* = 0.6744).

### 3.5. Safety Outcomes/Adverse Events

In the safety population, treatment-emergent adverse events (TEAEs) were reported in 9 of 113 pelubiprofen treatment periods (7.96%; 10 events) and 10 of 112 placebo treatment periods (8.93%; 11 events), with no significant between-treatment difference (*p* = 0.7361). All TEAEs were mild to moderate in severity. Adverse drug reactions (ADRs) were reported in one pelubiprofen treatment period (dyspepsia, 0.88%) and one placebo treatment period (hematochezia, 0.89%). One serious adverse event (SAE) occurred during the placebo treatment period (tonsillitis, 0.89%), whereas no serious adverse drug reactions were reported in either treatment period. The most frequently reported TEAE during the placebo treatment period was pyrexia (2/112, 1.79%); the remaining placebo-period TEAEs, each reported once, were hematochezia, nausea, vomiting, nasopharyngitis, tonsillitis, alopecia areata, headache, benign ovarian germ cell teratoma, and oropharyngeal pain. In the pelubiprofen treatment period, all TEAEs occurred once (1/113, 0.88%) and included dyspepsia, pyrexia, cystitis, eczema, hand dermatitis, dizziness, ventricular extrasystoles, hepatitis, myalgia, and vaginal hemorrhage. The only SAE was judged unrelated to study treatment.

## 4. Discussion

In this multicenter, randomized, double-blind, two-period crossover phase 3 trial, pelubiprofen, an NSAID with relatively selective cyclooxygenase-2 inhibitory activity, provided greater analgesic benefit than placebo in women with primary dysmenorrhea. Improvements were observed in both cumulative pain relief and pain intensity reduction during the first 8 h after dosing and were maintained through 12 h, supporting short-term symptom control. These findings align with the established role of NSAIDs in primary dysmenorrhea, in which suppression of prostaglandin-mediated uterine activity is central to pain relief [2,9,12]. In this context, comparative effectiveness across pharmacological and non-pharmacological options is still being clarified; notably, a protocol for a systematic review and network meta-analysis has been published to synthesize available evidence in primary dysmenorrhea [12,13].

Time-course analyses further supported rapid onset of analgesic benefit. In the present study, time-specific pain relief scores and pain intensity difference (PID) values favored pelubiprofen from 0.75 h onward through 8 h (Figure 3), indicating earlier separation from placebo and sustained advantage during the period captured in the time-course figure. Given that menstrual pain often peaks early after onset, earlier onset of relief is clinically relevant and may translate into improved functional outcomes and patient experience in routine care [2,12].

In addition to cumulative pain measures, patient-reported global satisfaction favored pelubiprofen. The distribution of Day 1 global evaluation at 12 h (Figure 5) showed higher ratings with pelubiprofen than placebo, and the proportion of patients rating treatment as “good” or better remained higher with pelubiprofen on subsequent days, with a statistically significant difference observed on Day 2. Patient-reported outcomes are clinically meaningful because they integrate perceived analgesic benefit with tolerability and overall treatment acceptability, which are important determinants of real-world adherence for episodic conditions such as dysmenorrhea [5,12]. These patient-reported outcomes help contextualize the statistical findings at the individual level; however, formal MCID- or NNT-based analyses were not prespecified, which limits the clinical interpretability of the cumulative summary endpoints.

The incidence of TEAEs was low and comparable between treatment periods, and all reported events were mild to moderate in severity. The only SAE occurred during the placebo treatment period and was considered unrelated to study treatment, and no serious adverse drug reactions were observed. Gastrointestinal adverse events, commonly associated with NSAID therapy, were infrequent in this short-term, as-needed dosing context. Pelubiprofen has been described as a 2-arylpropionic acid-derived prodrug with relatively selective COX-2 inhibitory activity, and these properties have been discussed as potential contributors to reduced GI ulcerogenicity compared with some non-selective NSAIDs in preclinical/clinical development literature [9]. These findings support the short-term tolerability of pelubiprofen in primary dysmenorrhea; nevertheless, the trial compared pelubiprofen with placebo only, meaning it does not provide direct comparative effectiveness or safety data versus standard NSAIDs commonly used in clinical practice [2,5,12]. Furthermore, because the study duration was limited to short-term, episodic use, longer-term safety outcomes and relative harm/benefit profiles compared to active standard care should be further evaluated in broader populations.

Several limitations should be considered. Secondary dysmenorrhea was excluded clinically, but standardized pelvic imaging was not mandated across sites; therefore, some participants with unrecognized secondary causes may have been included. Although the crossover design reduces between-subject variability and no significant carryover effect was detected, cycle-to-cycle variation in unmeasured within-subject factors such as stress, sleep, physical activity, diet, caffeine intake, and intercurrent illness may have introduced residual confounding. In addition, because the primary endpoints were cumulative summary measures and missing efficacy data were handled using LOCF, methodological bias cannot be excluded. The absence of formal MCID- or NNT-based analyses may also limit the clinical interpretability of the observed treatment differences. Finally, the external validity of our findings may be limited, as the study population was restricted to Korean women aged 19 to 44 years and excluded users of various concomitant medications. Future pragmatic trials or real-world observational studies are warranted to validate these findings in broader clinical populations.

## 5. Conclusions

Pelubiprofen at 45 mg provided superior short-term analgesic efficacy compared with placebo, with significant improvements in cumulative pain relief outcomes at 8 h and maintained differences at 12 h. Pelubiprofen was generally well tolerated, with a low incidence of adverse events comparable to placebo.

## Figures and Tables

**Table 1:** Baseline demographic and dysmenorrhea-related characteristics.

Characteristic	Sequence 1 (*n* = 60)	Sequence 2 (*n* = 60)	Total (*n* = 120)	*p*-Value
Age, mean (SD ^1^), y	30.97 (5.48)	29.17 (4.89)	30.07 (5.25)	0.060
Height, mean (SD), cm	161.82 (4.68)	161.86 (5.29)	161.84 (4.97)	0.969
Weight, mean (SD), kg	56.10 (7.01)	56.42 (6.16)	56.26 (6.57)	0.792
BMI ^2^, mean (SD), kg/m^2^	21.48 (3.01)	21.55 (2.32)	21.51 (2.67)	0.879
				
Number of dysmenorrhea episodes in the six menstrual cycles prior to screening, mean (SD)	5.65 (0.68)	5.62 (0.74)	5.63 (0.71)	0.798
Dysmenorrhea intensity, *n* (%)				0.609
Moderate	50 (83.33)	52 (86.67)	102 (85.00)	
Severe	10 (16.67)	8 (13.33)	18 (15.00)	

^1^ SD, standard deviation; ^2^ BMI, body mass index.

**Table 2:** Model-estimated LS means (±SE) for TOTPAR and SPID at 8 and 12 h and corresponding treatment contrasts in the mITT population.

Time Point/Parameter	Pelubiprofen 45 mg (*n* = 113)	Placebo (*n* = 111)	*p*-Value
8 h			
TOTPAR ^1^ (LS ^2^ mean ± SE ^3^)	22.17 ± 0.75	15.50 ± 0.76	
Difference in LS means (95% CI ^4^)	6.67 (4.82–8.53)		<0.0001
SPID ^5^ (LS mean ± SE)	10.00 ± 0.44	6.17 ± 0.45	
Difference in LS means (95% CI)	3.82 (2.71–4.94)		<0.0001
12 h			
TOTPAR (LS mean ± SE)	33.95 ± 1.14	25.41 ± 1.15	
Difference in LS means (95% CI)	8.54 (5.81–11.27)		<0.0001
SPID (LS mean ± SE)	15.34 ± 0.67	10.62 ± 0.67	
Difference in LS means (95% CI)	4.72 (3.13–6.31)		<0.0001

Values are based on analysis of covariance (ANCOVA) models. ^1^ TOTPAR, total pain relief; ^2^ LS, least squares; ^3^ SE, standard error; ^4^ CI, confidence interval; ^5^ SPID, summed pain intensity difference.

## Data Availability

The data presented in this study are not publicly available due to restrictions related to participant privacy and sponsor confidentiality. Data may be made available from the corresponding author upon reasonable request and with permission of the sponsor.
